# Descemet’s Membrane Detachment Following Inadvertent Intrastromal Injection of an Ophthalmic Viscoelastic Device

**DOI:** 10.7759/cureus.93383

**Published:** 2025-09-28

**Authors:** Adam Bharmal, Shankar Ramanathan, Balasubramanian Ilango, Abhijit A Mohite

**Affiliations:** 1 Ophthalmology, The Royal Wolverhampton NHS Trust, Wolverhampton, GBR

**Keywords:** glaucoma surgery, intracameral c3f8, mitomycin c (mmc), ophthalmic viscoelastic device, postoperative hypotony, primary open angle glaucoma, trabeculectomy bleb

## Abstract

Hypotony is a well-documented postoperative complication of glaucoma filtration surgery. Management may include the injection of an ophthalmic viscoelastic device (OVD) into the anterior chamber. This case report describes a 45-year-old woman with juvenile-onset open-angle glaucoma who developed clinical hypotony after trabeculectomy surgery augmented with mitomycin-C. An intracameral injection of Healon5® (Johnson & Johnson Vision, USA) was attempted to restore physiological intraocular pressure (IOP); however, Healon5® was inadvertently introduced into the corneal stroma, causing a localised Descemet’s membrane detachment (DMD). Following non-resolution with observation and attempted surgical evacuation, an intracameral air tamponade successfully addressed the DMD. An intracameral air tamponade may provide a quicker alternative to prolonged observation, particularly for highly cohesive OVDs such as Healon5®.

## Introduction

Hypotony is a well-known postoperative complication of glaucoma filtration surgery and may be associated with sight-threatening complications such as hypotony maculopathy, choroidal detachment, and suprachoroidal haemorrhage [1]. In the context of glaucoma filtration surgery, it often results from either over-filtration or ciliary body shutdown. Incidence of hypotony following trabeculectomy varies in the literature from as low as 1.5% [2] up to 33% [3]. Ophthalmic viscoelastic devices (OVDs) are commonly employed substances in ophthalmic surgery owing to their ability to create and maintain spaces within the eye. The injection of OVDs such as Healon5® (Johnson & Johnson Vision, USA) is a recognised temporising measure in restoring physiologic intraocular pressure (IOP), particularly in the context of a shallow anterior chamber [4,5].

We report a case of inadvertent intrastromal injection of Healon5® causing a localised Descemet’s membrane detachment (DMD), managed successfully with an intracameral air tamponade. 

## Case presentation

A 45-year-old woman with a history of juvenile-onset open-angle glaucoma was referred to the hospital eye services four years ago. Initially, she underwent 180 degrees of inferior selective laser trabeculoplasty in the right eye, but this failed to control her IOP. Consequently, she underwent a right eye trabeculectomy augmented with mitomycin C. Postoperatively, she developed iris incarceration within the trabeculectomy ostium, which required surgical release.

One week after this procedure, she developed clinical hypotony in the right eye, with an IOP of 2 mmHg on Goldman applanation and choroidal effusions. To manage this, she consented to an intracameral injection of OVD.

During the procedure, 0.1 ml of Healon5® (2.3% sodium hyaluronate) was introduced into the eye using a 30-gauge needle (Beaver-Visitec International, USA) via a long inferotemporal corneal tunnel. Due to a shallow anterior chamber and low IOP, it was difficult to ensure the tip of the needle had fully entered the eye, resulting in the inadvertent injection of Healon5® into the corneal stroma. This caused a localised DMD in the paracentral inferior cornea measuring 2.5 mm vertically by 2.3 mm horizontally. A small area of well-demarcated corneal stromal haze was observed two days later. The central cornea corresponding to the visual axis was spared, and the anterior chamber was well formed with no intraocular inflammation. Given her preserved central vision of 6/9, the decision was made to monitor the condition for spontaneous resolution.

After two weeks of observation with no improvement in the DMD, a surgical attempt was made to evacuate the trapped Healon5®. A stromal pocket was created at the detachment site using a 23-gauge microvitreoretinal (MVR) blade (Alcon Laboratories, Inc., USA), and an intracameral tamponade with OVD was performed through a separate paracentesis incision. However, the high viscosity of Healon5® limited the effectiveness of this procedure, as only a small amount of viscoelastic could be expressed through the stromal pocket.

Following an additional four weeks of observation with no significant improvement in the size of the DMD, another attempt was made to remove the trapped Healon5®. On this occasion, an intracameral air tamponade was used. An air bubble was introduced into the anterior chamber via a paracentesis and left in place for 10 minutes before being washed out with a Simcoe cannula (Beaver-Visitec International, USA) and balanced salt solution.

**Figure 1:**
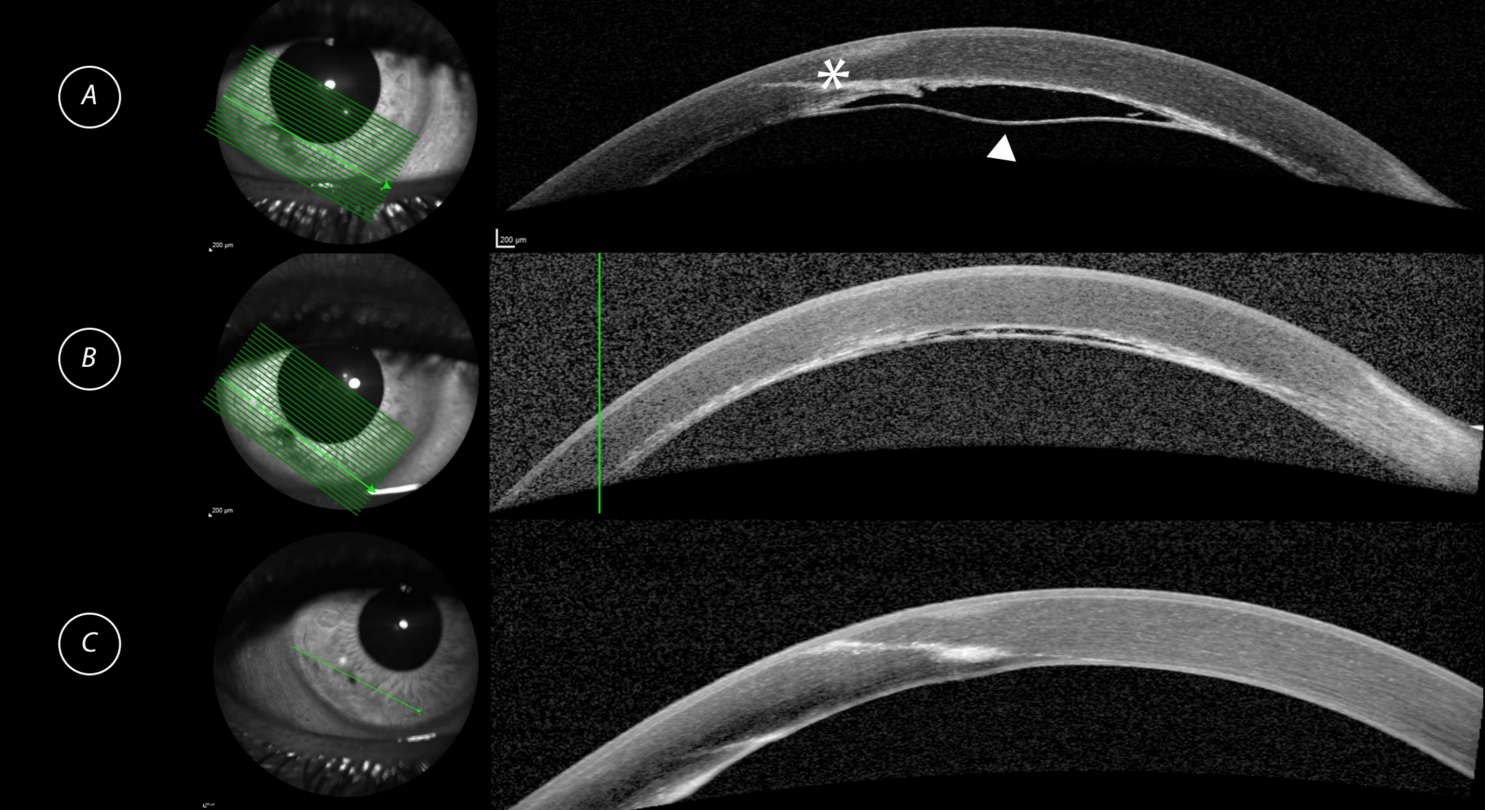
AS-OCT imaging of the inferior DMD at various timepoints (A) Inferior corneal DMD (white arrowhead) one week prior to air tamponade. The long intrastromal needle track is demonstrated (*). (B) Immediately post air tamponade demonstrating a marked improvement of the inferior corneal DMD. A hyper-reflective interface is noted at the junction between Descemet’s membrane and stroma. (C) Six months post air tamponade demonstrating complete resolution of the inferior corneal DMD. AS-OCT: anterior segment optical coherence tomography; DMD: Descemet’s membrane detachment

Immediately after this procedure, there was a marked improvement in the size of the DMD. Figure 1a demonstrates the inferior DMD on an anterior segment optical coherence tomography (AS-OCT) image. Figure 1b demonstrates a marked improvement in the DMD immediately following intracameral air tamponade. Further imaging performed at six months post air tamponade, shown in Figure 1c, revealed complete resolution of the DMD. At six months postoperatively, her best-corrected visual acuity was 6/9, and her IOP was 7 mmHg.

## Discussion

Clinical hypotony is an uncommon consequence of glaucoma filtration surgery, with postoperative incidence after trabeculectomy ranging from 1.5-33% [2,3]. Robust strategies for managing hypotony are necessary, given the potential complications such as choroidal detachment and suprachoroidal haemorrhage [1]. The use of OVD injected into the anterior chamber is regarded as a generally safe and effective measure that can be performed in a clinic or theatre setting [4,5].

In this case, OVD was injected into the anterior chamber with the use of a 30-gauge needle through a long corneal tunnel. The advantage of this method over the creation of a paracentesis is that the procedure can often be performed safely at the slit lamp without the need for an operating theatre setup. This method may also minimise any further shallowing of the anterior chamber. Precise titration of the volume of the OVD injected into the anterior chamber can help avoid overfill and subsequent spikes in IOP. Our standard protocol is to inject 0.1 ml of OVD followed by a further 0.05-0.1 ml if the anterior chamber has not sufficiently deepened and the IOP has not started to increase immediately afterwards.

Complications from an intracameral injection of OVD are sparsely reported in the literature. While there is a theoretical risk of exogenous endophthalmitis, there are currently no case reports in support of this. One case report demonstrated an extreme increase in IOP following an intracameral injection of Healon5® in a patient with a XEN® gel stent (AbbVie, USA) in situ, necessitating an anterior chamber washout [6].

There are very few reports in the literature of a DMD induced by inadvertent intrastromal injection of OVD [7-10]. Management strategies vary, including observation with spontaneous resolution. For example, a four-year-old girl who had Viscoat® (3% sodium hyaluronate, 4% chondroitin sulfate) (Alcon Laboratories, Inc., USA) injected inadvertently into the cornea experienced a DMD, which resolved spontaneously within three months [10]. Another case involved the spontaneous recovery of a DMD after Healon GV® (1.8% sodium hyaluronate) (Johnson & Johnson Vision, USA) was injected, resolving at six weeks [7]. In the case of a large central DMD following inadvertent intrastromal injection of OVD, one author describes the use of three full-thickness 10-0 nylon sutures to secure the Descemet’s membrane. The DMD subsequently resolved at two months of follow-up, and the sutures were removed [9].

The duration of DMD in cases managed conservatively ranged from six weeks to seven months. The viscosity of the injected OVD likely influences the spontaneous clearance rate. Healon5® has the highest cohesive index of the commonly used OVDs for ophthalmic surgery and, as such, may have the slowest spontaneous clearance rate [11]. The mechanism by which OVDs are spontaneously cleared from the cornea is not well understood. Suggestions include microtears in Descemet’s membrane allowing efflux of OVD [7]. An immune response may also be responsible, as demonstrated in a histologic study of enucleated rabbit eyes, where a macrophage invasion ingested intrastromal OVD [12].

The visual prognosis has been generally positive following spontaneous or surgical resolution of the DMD in all of the cited case reports [7-10]; however, the often long recovery period of up to six months may have a significant impact on the patient in question, particularly in cases of large central DMDs. 

An intracameral air tamponade, as demonstrated in this case, may provide a more rapid surgical solution. In this scenario, an air bubble was introduced into the anterior chamber and left in place prior to being washed out. A possible limitation of this method is the short duration of tamponade applied to the DMD and the subsequent risk of non-resolution. Alternate methods include leaving the air bubble in situ with the patient posturing in a supine position to maximise the tamponading effect on the inferior DMD. Expansile gases such as perfluoropropane (C3F8) or sulfur hexafluoride (SF6) could also be options, similar to the advantage of providing a longer period of tamponade. However, a major limitation would be the necessity of an iridectomy to prevent pupillary block [13].

## Conclusions

This is a single case report demonstrating the successful reattachment of a DMD secondary to an inadvertent intrastromal injection of OVD with the use of an air tamponade. Though several cases have demonstrated spontaneous resolution of a DMD in similar circumstances, this can take several months, especially where highly cohesive OVDs such as Healon5® have been employed. The use of an intracameral air tamponade may accelerate this process and could therefore be a useful intervention in the management of similar complications. Further reports would be required to establish the safety profile and repeatability of this intervention. 
